# Editorial: Molecular mechanisms and clinical studies of multi-organ dysfunction in sepsis associated with pathogenic microbial infection

**DOI:** 10.3389/fcimb.2026.1789175

**Published:** 2026-02-13

**Authors:** Yun Li, Qinghe Yan, Shuoyan Dong, Lina Zhao

**Affiliations:** 1 Department of Anesthesiology, The Second Hospital of Tianjin Medical University, Tianjin, China; 2 Department of Critical Care Medicine, Tianjin Medical University General Hospital, Tianjin, China

**Keywords:** acute kidney injury, biomarkers, immune-metabolic interactions, oxidative stress, predictive models, sepsis

Recent studies published in Frontiers in Cellular and Infection Microbiology have significantly advanced our understanding of sepsis, a life-threatening condition characterized by dysregulated host responses to infection. These five articles explore diverse aspects of sepsis, including novel biomarkers, immune-metabolic interactions, and predictive scoring systems, offering new insights into early diagnosis, risk stratification, and therapeutic interventions.

## 1 Key findings

### 1.1 Predictive models for sepsis-induced myocardial injury

Liu et al. evaluated the prognostic value of Sequential Organ Failure Assessment (SOFA) and Acute Physiology Score III (APSIII) in patients with sepsis-associated myocardial injury. Their retrospective analysis of the MIMIC-IV database demonstrated that both scores independently predicted 28-day mortality, with SOFA (AUC = 0.685) and APSIII (AUC = 0.683) outperforming traditional biomarkers like procalcitonin (PCT) and C-reactive protein (CRP). This highlights the importance of multi-organ dysfunction assessment in sepsis management.

### 1.2 Oxidative stress dynamics in sepsis: a single-cell perspective

Xu et al. employed single-cell RNA sequencing to investigate oxidative stress (OS) in sepsis, identifying LILRA5+ macrophages as key drivers of early OS surge. The study revealed that LILRA5 silencing reduced reactive oxygen species (ROS) levels *in vitro*, suggesting a potential therapeutic target. This work provides a high-resolution view of sepsis immunopathology and underscores the role of macrophage-mediated inflammation in disease progression.

### 1.3 Neutrophil-to-prognostic nutritional index ratio as a novel biomarker

Lou et al. introduced the neutrophil/PNI ratio (NPR) as a prognostic marker for ICU mortality in sepsis. Their analysis of MIMIC-IV data showed a J-shaped association between NPR and mortality, particularly in elderly and obese patients. NPR integrates inflammatory and nutritional status, offering a simple yet effective risk assessment tool.

### 1.4 Heparin-Binding Protein and IL-6 in severe pneumonia with sepsis

Wei et al. compared HBP and IL-6 with conventional biomarkers in predicting 28-day mortality in sepsis secondary to severe pneumonia. IL-6 exhibited the highest discriminative power (AUC = 0.80), surpassing CRP and lactate. Additionally, IL-6 was significantly elevated in bloodstream infections, supporting its role in early bacteremia detection.

### 1.5 Creatinine-to-albumin ratio in sepsis-associated AKI

Another study by Lou et al. explored CAR as a predictor of 30-day mortality in sepsis-induced acute kidney injury (AKI). A threshold of CAR ≥1.2 mg/dL was associated with increased ICU mortality (HR = 1.61), independent of metabolic and hemodynamic confounders. This ratio, easily calculated from routine lab tests, enhances risk stratification in critically ill patients.

## 2 Clinical implications and future directions

These studies collectively emphasize:

1. Improved risk stratification: SOFA, APSIII, NPR, and CAR provide simple yet robust tools for mortality prediction.
2. Novel biomarkers: LILRA5, IL-6, and HBP offer mechanistic insights and potential therapeutic targets.
3. Heterogeneity in sepsis: Single-cell analyses reveal subtype-specific immune responses, paving the way for precision medicine.

Future research should focus on:

1. Prospective validation of these models in diverse populations.
2. Integration of multi-omics data (e.g., metabolomics, proteomics) to refine predictive accuracy.
3. Interventional trials targeting identified biomarkers (e.g., anti-LILRA5 therapies).

## 3 Conclusion

These findings significantly enhance sepsis management by improving early diagnosis, risk assessment, and personalized treatment strategies. The integration of computational models, biomarkers, and advanced sequencing techniques holds promise for reducing sepsis-related mortality worldwide.

